# The Endocrine Function of the Heart: Physiology and Involvements of Natriuretic Peptides and Cyclic Nucleotide Phosphodiesterases in Heart Failure

**DOI:** 10.3390/jcm8101746

**Published:** 2019-10-21

**Authors:** Claire Lugnier, Alain Meyer, Anne Charloux, Emmanuel Andrès, Bernard Gény, Samy Talha

**Affiliations:** 1 Institute of Physiology, FMTS-EA 3072, Faculty of Medicine, University of Strasbourg, 11 Humann Street, 67000 Strasbourg, France; claire.lugnier@unistra.fr (C.L.); alain.meyer1@chru-strasbourg.fr (A.M.); anne.charloux@chru-strasbourg.fr (A.C.); emmanuel.andres@chru-strasbourg.fr (E.A.); bernard.geny@chru-strasbourg.fr (B.G.); 2 Department of Physiology and Functional Explorations, New Civil Hospital, University Hospitals of Strasbourg, 1 Place de l’Hôpital, CEDEX 67091 Strasbourg, France; 3 Department of Internal Medicine and Metabolic Diseases, Medical Clinic B, Civil Hospital, University Hospitals of Strasbourg, 1 Place de l’Hôpital, CEDEX 67091 Strasbourg, France

**Keywords:** heart failure, cyclic nucleotide phosphodiesterase, natriuretic peptides, ANP, BNP, biomarkers, clinical management

## Abstract

Besides pumping, the heart participates in hydro-sodium homeostasis and systemic blood pressure regulation through its endocrine function mainly represented by the large family of natriuretic peptides (NPs), including essentially atrial natriuretic (ANP) and brain natriuretic peptides (BNP). Under normal conditions, these peptides are synthesized in response to atrial cardiomyocyte stretch, increase natriuresis, diuresis, and vascular permeability through binding of the second intracellular messenger’s guanosine 3′,5′-cyclic monophosphate (cGMP) to specific receptors. During heart failure (HF), the beneficial effects of the enhanced cardiac hormones secretion are reduced, in connection with renal resistance to NP. In addition, there is a BNP paradox characterized by a physiological inefficiency of the BNP forms assayed by current methods. In this context, it appears interesting to improve the efficiency of the cardiac natriuretic system by inhibiting cyclic nucleotide phosphodiesterases, responsible for the degradation of cGMP. Recent data support such a therapeutic approach which can improve the quality of life and the prognosis of patients with HF.

## 1. Introduction

Over the past decades, much work supported the hypothesis that the heart not only has a mechanical but also an endocrine function. The existence of hormonal systems in the heart tissue (i.e., the biochemical components necessary for hormone synthesis) provides the heart with the ability of participating in cardiovascular homeostasis and metabolism in addition to its pumping function. These cardiac hormones may affect remote tissues (endocrine function of the heart) and/or have local effects (paracrine and autocrine actions) [1,2], which may influence the action of cyclic nucleotide phosphodiesterases (PDEs).

Proteins secreted from cardiomyocytes, cardiac fibroblasts, endothelial cells, and smooth muscle cells in response to changes in the cardiac environment are called “cardiokine”, and specific cardiomyocytes-derived peptides are referred to as “cardiomyokines” [3]. Although other cardiac hormones have been described, such as adrenomedullin, endothelin 1, secreted phospholipase A_2_, follistatin-like 1, chromogranin A, fibroblast growth factors, osteocrin and cardiomyocytes proteins using extra-cellular vesicles able to modulate the adrenergic system (for review, see References [3,4]). Here, we describe mainly natriuretic peptides (NPs) and, more specifically, atrial natriuretic peptide (ANP) and brain natriuretic peptide (BNP). Indeed, these NPs play a major role in cardiac endocrine function through the second messenger, guanosine 3′,5′-cyclic monophosphate (cGMP), and interactions with PDEs. We will review both natriuretic peptides physiology and pathology, focusing on their roles in cardiac failure and on the implications of the PDEs family, which might open new therapeutic approaches.

## 2. Physiology

### 2.1. The Family of Natriuretic Peptides

In 1981, De Bold [5] demonstrated the synthesis by atrial myocytes of the atrial natriuretic factor (ANF), which was then called atrial natriuretic peptide (ANP). The brain natriuretic peptide or type B natriuretic peptide (BNP), initially discovered in 1988 in pig brain [6], is in fact mainly located in atrial cardiomyocytes. A third peptide belonging to this family, the C-type natriuretic peptide (CNP), is located at a relatively low level in healthy individuals in the central nervous system, chondrocytes, and in the cardiovascular system (endothelial cells, cardiomyocytes, and fibroblasts). Cardiac gene expression and plasma levels of CNP are increased in patients with heart failure (HF) and associated with a high-risk phenotype. Endogenous roles of CNP include the control of vascular tone, angiogenesis, coronary blood flow modulation, smooth muscle and endothelial cell proliferation, cardiac fibrosis and hypertrophy, and leukocyte activation (for review see Reference [7]). Urodilatin, the fourth member of this family, is synthesized and secreted by the distal tubule of the nephrons and is detected only in the urine. Urodilatin interacts in a paracrine way with sensitive amiloride sodium channels to promote diuresis and natriuresis and, thus, participates in hydro-sodium regulation [8].

Within the NP family, we describe the biochemical, physiological, and pathophysiological characteristics of ANP and BNP.

### 2.2. Structure, Synthesis, and Secretion

These peptides are all synthesized in the form of pro-hormones, mainly produced by cardiovascular, cerebral, and renal tissues. They are characterized by a common 17 amino acid ring structure closed by a disulfide bond between two cysteines essential for their biological activity. The ANP and BNP are encoded by distinct genes, *Nppa* and *Nppb*, respectively, but located on the same chromosome 1. Their expressions in cardiomyocytes has been mainly identified at the atrial level [9,10]. Other cells also synthetize and secrete A- and BNP, such as fibroblasts, endothelial cells, immune cells (neutrophils, T cells, and macrophages), embryonic progenitor hematopoietic stem cells, satellite muscle cells, and heart precursors [11].

Human pre-proANP is composed of 151 amino acids (aa), then cleaved into 126 aa proANP, the main form of storage in atrial granules. Rapidly, proANP is cleaved upon secretion by corin, a trans-membrane serine endoprotease, to form the biologically active ANP (28 aa) and the biologically inactive N-terminal fragment (NT-proANP, 98 aa). ANP is also present in other tissues such as the heart ventricles and kidney but at lower concentrations. Urodilatin results from an alternative cleavage process of the 32 aa ANP by an unknown protease and plays a role in regulating hydro-sodium renal excretion by a paracrine effect [8]. In response to variations in atrial volume (thus inducing tissue stretching), rather than a change in atrial pressure [12], ANP, with a plasma half-life of 3 to 5 min, is mainly secreted from a pool of peptides previously synthesized and stored in granules in mainly atrial cardiomyocytes.

Human BNP is initially synthesized as 134 aa pre-prohormone, cleaved into 108 aa proBNP cardiomyocytes, which in turn will be cleaved by corin or furin into 32 aa biologically active BNP, and 76 aa biologically inactive NT-proBNP. ProBNP, BNP, and NT-proBNP are secreted and can be measured in plasma. Although BNP is mainly co-stored with ANP in atrial granules in healthy individuals, it can also be synthesized in ventricular cardiomyocytes at significant levels in HF conditions, but will not be stored in granules in the latter case. The regulation of BNP gene transcription and excretion is essentially based on the degree of stretching of the myocardial wall resulting from volume overload and/or increased transmural gradient [10]. The half-lives of BNP and NT-proBNP are approximately 20 min and 120 min, respectively.

### 2.3. Receptors and Clearance

There are three types of NP receptors: natriuretic peptide receptors of types A (NPR-A) and B (NPR-B), also known as particulate and biologically active guanylyl cyclases, and the type C receptor (NPR-C) acting as a clearance receptor. The NPs exert their physiological effects essentially by binding to the high-affinity receptors, NPR-A for ANP and BNP, NPR-B for CNP. The binding potential of NPs to their receptors is as follow: NPR-A = ANP ≥ BNP > CNP; NPR-B = CNP ≥ ANP > BNP; NPR-C = ANP ≥ CNP > BNP [13].

These receptors, coupled with guanylyl cyclase, are widely distributed throughout the body, including the heart, brain, kidneys, adrenals, lungs, terminal ileum, aorta, fibroblasts, and adipocytes. The NPR-A and NPR-B are composed of three domains: an extracellular segment of approximately 450 aa recognizing and fixing the NP, a short trans-membrane segment, and an intracellular region of about 570 aa composed of a pseudokinase or kinase homology domain, a dimer domain and the domain of guanylyl cyclase activity responsible for the synthesis of cGMP, the second messenger of the NPs.

NPR-C is strongly present in the kidneys at the glomerular and vascular levels, in the vascular wall, adrenals, heart, mesentery, placenta, lungs, and brain. Its extracellular domain is approximately 30% identical to that of NPR-A and NPR-B, but this receptor contains only 37 intracellular aa without a guanylyl cyclase activity domain, but with a potential signaling function [14].

Two main mechanisms participate in the clearance of cardiac peptides: a cellular mechanism through membrane internalization of the ligand-NPR-C complex, followed by a lysosomal hydrolysis of NP and a recycling of the NPR-C on the cell surface [15], and an enzymatic mechanism through the action of a cleavage proteolytic enzyme, the neprilysin (NEP). However, growing evidence supports that other proteases play a role in the clearance of NPs. Meprin A is shown to be involved in the initial N-terminal cleavage of BNP, and meprin A and NEP are thought to work together in the clearance of BNP. In addition, NPs are also inactivated by the action of dipeptidyl peptidase-4 (DPP IV) and insulin degrading enzyme (IDE), belonging to the metalloproteinase family [16]. In addition to these two clearance mechanisms, there is also urinary excretion of NP [17].

### 2.4. Signaling Pathways

The binding of agonists to NPR-A and NPR-B induces a conformational change that removes the inhibition exerted by the pseudokinase domain at the enzyme site, allowing intracellular cGMP synthesis from guanosine triphosphate with, consequently, an increase in circulating and urinary cGMP levels. The intracellular targets of cGMP are cGMP-dependent protein kinases G of types I and II (PKG-I and II), cyclic nucleotide-gated channels (CNGC), and certain specific PDEs that directly control the level of cyclic nucleotides and, more particularly, that of cGMP [18,19].

#### 2.4.1. Protein Kinase G of Types I and II

The vasorelaxing effect of NPs on vascular smooth muscles is partly mediated by PKG-I, abundant in particular in cardiomyocytes and the vascular system. It reduces the presence of intracellular Ca^2+^ by several synergistic actions: increase in Ca^2+^ membrane output, decrease in Ca^2+^ membrane input, sequestration of Ca^2+^ in the sarcoplasmic reticulum, and decrease in Ca^2+^ mobilization. All actions decrease the sensitivity of the contractile elements to Ca^2+^ and promote muscle relaxation. These synergistic actions of PKG-I are carried out via the phosphorylation of the voltage-dependent Ca^2+^ channels, K^+^/Ca^2+^-dependent channels (BK and K_ATP_ channels), the transmembrane Ca^2+^-ATPase pump, the inositol triphosphate receptor, and phospholambans located on the sarcoplasmic reticulum membrane [20].

Protein Kinase G of type II is a membrane enzyme absent from the cardiovascular system but present in the kidney, where it exerts a proximal tubular action by inhibiting the reabsorption of Na^+^ by the Na^+^/H^+^ exchanger (NHE3) and an action on juxta-glomerular cells by inhibiting renin secretion and, thus, angiotensin II and aldosterone synthesis, resulting in a distal decrease in water and sodium reabsorptions [21].

More than a clearance receptor, and although lacking guanylyl cyclase and kinase activities, NPR-C is now considered biologically active. Indeed, combined with an intracellular domain of activation of the α subunit of the G_i_ protein, activated NPR-C would inhibit adenylyl cyclase activity and decrease the level of intracellular adenosine 3′,5′-cyclic monophosphate (cAMP), which modulates phospholipase C (PLC), extracellular signal-related kinase (ERK) 1/2, and protein kinase B (Akt). Thus, its stimulation would inhibit the proliferation of vascular smooth muscle cells through the mitogen-activated protein kinase (MAPK) pathway and phospho-inositol 3 kinase (PI3-kinase) pathway [7]. More generally, it was suggested that the effects attributed to ANP, BNP or CNP, but not explained by an increase in cGMP, could ultimately be attributed to the stimulation of NPR-C by these NPs. These effects would concern the inhibition of aldosterone, renin, and vasopressin secretions by ANP, as well as the anti-proliferative effect of ANP and BNP. The biological activation of NPR-C is thought to be involved in many diseases such as high blood pressure, obesity, coronary heart disease or HF [22]. In this context, it is clearly established that different dysfunctions of the PDEs are also involved in these pathologies [19].

#### 2.4.2. Cyclic Nucleotide Phosphodiesterases (PDEs)

**Figure 1:**
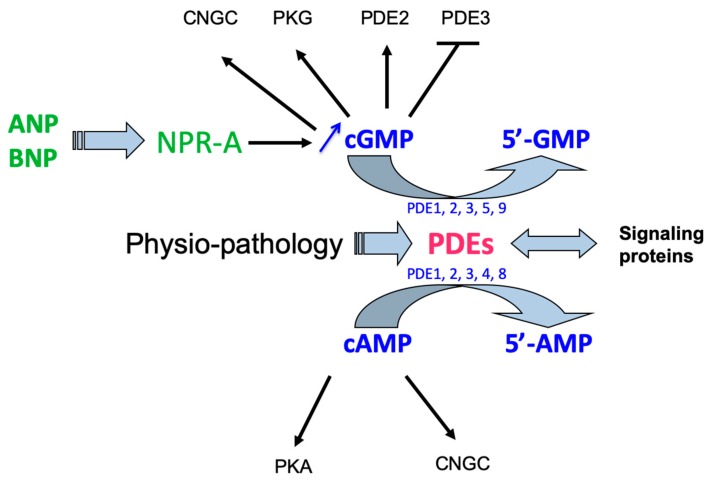
Interactions between natriuretic peptides and cyclic nucleotide pathways. By stimulating the NPR-A receptor, the natriuretic peptides ANP and BNP increase cGMP formation from guanosine triphosphate (GTP). This increase in cGMP, in addition to cGMP-protein kinase activation (PKG), might also activate PDE2 (which hydrolyses both cAMP and cGMP), inhibits PDE3 (which hydrolyses mainly cAMP, but also cGMP with a higher affinity), whereas it increases the activity cyclic nucleotide gated channels (CNGC). Below, receptor regulation modulating intracellular cyclic nucleotide levels and cyclic nucleotide phosphodiesterases (PDEs) very quickly hydrolyze cAMP and/or cGMP, controlling therefore the protein-kinase dependent phosphorylations (PKA and PKG), as well as CNGC in normal and physio-pathologic conditions. Reciprocally, PDE activity might be regulated by phosphorylation and by gene expression. In some physiopathologies, some PDE subtypes might be specifically altered, notably in cardiovascular diseases, opening new therapeutic approaches (see Section 2.4. and Reference [12]). 5′ AMP, 5′ adenosine monophosphate; cAMP, adenosine 3′,5′-cyclic monophosphate; ANP, atrial natriuretic peptide; BNP, brain natriuretic peptide; CNGC, cyclic nucleotide-gated channels; 5′ GMP, 5′ guanosine monophosphate; cGMP, guanosine 3′,5′-cyclic monophosphate; NPR-A, natriuretic peptide receptor of type A; PDE, cyclic nucleotide phosphodiesterase; PKA, protein kinase A; PKG, protein kinase G.

Natriuretic peptides act at the heart level by stimulating the synthesis of cyclic nucleotides such as cGMP. PDEs, responsible for their hydrolysis govern physiological and hormonal responses under normal and pathological conditions by controlling the signalosome at the phosphorylation cascades and the expression of genes dependent on cyclic nucleotides. It is therefore important not to neglect this important path of cGMP regulation mediated by the NPs (Figure 1).

Cyclic nucleotide phosphodiesterases represent an enzymatic superfamily consisting of 11 gene families (PDE1 to PDE11). Each family includes 1 to 4 distinct genes, representing a set of more than 20 genes in mammals that code for more than 100 different proteins or iso-enzymes (for reviews, see References [19,23,24,25]. Cyclic nucleotide phosphodiesterases generally exist in the form of dimers. Their monomeric structures have a common structure of three distinct domains. The N-terminal regulatory domain characterizes each family and its variants. The catalytic domain, consisting of about 350 aa, is relatively preserved.

The multiplicity of biochemical and structural properties of PDEs, their tissue specificities, subcellular distributions, transcriptional and post-transcriptional regulations allow target-altered PDE iso-enzymes during a pathology, in order to avoid or reduce adverse effects induced by non-specific treatments.

With regard to cardiovascular pathologies, the intracellular signaling alterations induced by the deregulation of mainly cardiac PDEs could explain some therapeutic difficulties encountered. The main cardiac PDEs are as follows (Table 1):

**PDE1:** Mainly cytosoluble, this enzyme is present in the heart [26] and vascular smooth muscle where it was purified and characterized for the first time [27]. Both PDE1A and PDE1B preferentially hydrolyze cGMP, while PDE1C hydrolyzes cAMP and cGMP. Due to the fact of its properties, it participates in the regulation of cyclic nucleotides and calcium response at the cardiovascular level [28].

**PDE2:** Hydrolyzes both cAMP and cGMP, it plays a major role in the feedback of basal cGMP and cAMP in response to an increase in cGMP, particularly following the production of NO, ANP, and BNP. PDE2 is present in the heart [26], endothelial cells [29], and absent in the vascular smooth muscle [27].

**PDE3:** Has a higher affinity for cGMP than for cAMP. Due to the fact of its enzymatic properties in the heart, PDE3 participates differently from PDE2 in the interaction between the regulatory pathways of cAMP and cGMP [26], and vascular muscle [30]. PDE3A is mainly present in the heart and vascular muscle and at the level of endothelial cells. Its specific inhibition increases cardiac strength while inducing vascular relaxation. Milrinone is the first PDE3 inhibitor used in HF (Corotrope^®^, [31]).

**PDE4:** The family of PDE4, represents the largest family. It specifically hydrolyzes cAMP. Its insensitivity to cGMP differentiates PDE4 from PDE3 [32]. PDE4 is present in the heart, vascular smooth muscle, endothelial cells, and immunocytes.

**PDE5:** This was characterized and discovered as cGMP-PDE in 1986 from vascular smooth muscle. PDE5 is present in the heart, vascular smooth muscle, and endothelium. A specific inhibitor of PDE5, sildenafil (Viagra^®^, [33]) was used in the treatment of erectile dysfunction [34]. This use in humans has demonstrated the beneficial effect of PDE5 inhibitors in HF [35,36] and in the treatment of pulmonary arterial hypertension (PAH) [37].

**PDE8:** Hydrolyses specifically cAMP and participates in the control of cardiac function at the excitation–contraction coupling of cardiomyocytes in the ventricle [38].

**PDE9:** Hydrolyses specifically cGMP, and it has been characterized at the cardiac level where its regulation is increased in dilated cardiomyopathies [39].

### 2.5. Physiological Effects of ANP and BNP

**Figure 2:**
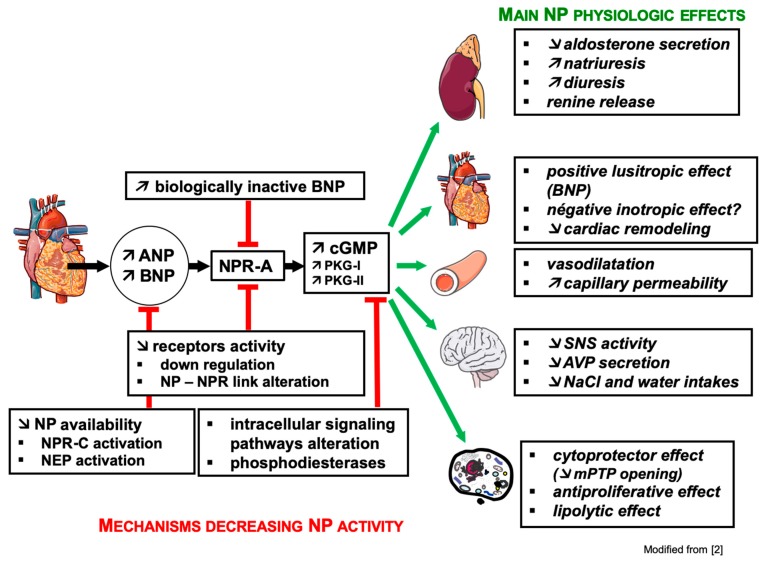
The main physiological effects of cardiac natriuretic peptides (NPs) and mechanisms reducing their activities. ANP, atrial natriuretic peptide; AVP, arginine vasopressin; BNP, brain natriuretic peptide; cGMP, guanosine 3′,5′-cyclic monophosphate; NaCl, sodium chloride; NEP, neutral endopeptidase; NPR-A, natriuretic peptide receptor of type A; NPR-C, natriuretic peptide receptor of type C; PKG-I/II, protein kinase G of types I/II; mPTP, mitochondrial permeability transition pore; SNS, sympathetic autonomous nervous system.

Due to the wide distribution of NPR-A, biological effects of NPs are numerous and mainly promote a decrease in blood volume and blood pressure (Figure 2).

#### 2.5.1. Renal Effects

The potent natriuretic and diuretic effects of ANP and BNP are mediated mainly by PKG-II present in the epithelial cells of the different segments of the nephron. These effects are related to an increased glomerular ultrafiltration rate and filtration fraction, inhibition of the amiloride-sensitive sodium apical channel, and basal-lateral Na^+^/K^+^ adenosine triphosphatase pump, promoting a decrease in sodium reabsorption at the collection tube (main mechanism) therefore increasing urinary sodium excretion [40]. The increase in glomerular filtration rate and filtration fraction results from the dilation of the afferent arterioles associated with the constriction of efferent arterioles (mediated by PKG-I), leading to an increase in the glomerular capillary hydrostatic pressure. These effects promote ultrafiltration and also contribute to reduced sodium gradient and water reabsorption [41]. In addition, ANP inhibits the renin–angiotensin–aldosterone system (RAAS) and, thus, reduces the transport of sodium and water induced by angiotensin II in the proximal tubule, renin secretion by juxtaglomerular granular cells, and aldosterone synthesis by the adrenal glands [14,42].

#### 2.5.2. Cardiovascular Effects

Although this effect does not strictly reflect an endocrine action but rather an autocrine one, BNP has a positive lusitropic effect by promoting myocardial relaxation and, thus, ventricular filling [43].

At the vascular level, two main mechanisms account for the action of ANP and BNP—the relaxation of vascular smooth muscle tone and, therefore, peripheral resistance by stimulating nitrogen monoxide (NO) synthesis and inhibition of RAAS on the one hand, and the increase in capillary permeability leading to an increase in hematocrit, on the other hand [44]. Increased capillary permeability appears to be the main effect of NPs under physiological conditions.

#### 2.5.3. Effects on Neuro-Hormonal Systems

The ANP modulates the activity of the autonomic nervous system. It reduces the activity of baroreceptors and cardiac and pulmonary chemoreceptors, thus inhibiting the sympathetic effector pathways to the heart. This reduction in sympathetic activity and increased vagal activity leads to a decrease in heart rate and output [3,45]. In addition, the inhibition of renal sympathetic activity by ANP and BNP also promotes decreased renin release and sodium reabsorption. Both ANP and BNP contribute to the reduction of blood volume by inhibiting thirst, salt intake [45], and angiotensin II-induced secretion of arginine vasopressin in the pituitary gland, thereby inhibiting water reabsorption through type 2 aquaporins in the renal main collector [46]. 

#### 2.5.4. Cellular Effects

ANP inhibits the proliferation of vascular smooth muscle cells and angiotensin II-induced hypertrophy under cell culture conditions [47]. BNP also inhibits cardiac remodeling by antifibrotic effects [48].

In addition, NPs regulate the processes of differentiation and cardiomyocyte proliferation during embryonic life, leading to high levels of BNP during gestation in the embryonic heart, together with a high plasma BNP level at birth which then gradually decreases to stabilize around the age of 10 years [49,50,51].

NPs also have cytoprotective properties. They limit the size of an infarction caused by coronary ligation-reperfusion in a heart model of isolated rats. This preconditioning phenomenon would be associated with an increase in cGMP and would involve the opening of mitochondrial K_ATP_ channels [52]. Anti-apoptotic [53] and anti-oxidant [54] properties of BNP have also been identified but remain controversial [55,56].

In skeletal muscle, recombinant BNP prevents the deleterious effects of ischemia-reperfusion by reducing mitochondrial respiratory chain dysfunction, reactive oxygen species (ROS) production, and apoptosis, possibly involving the opening of mitochondrial K_ATP_ channels [57].

#### 2.5.5. Effects on Adipose Tissue

Through actions on perilipin A and HSL (hormone sensitive lipase) secondary to the activation of the cGMP/PKG-I pathway, NPs regulate the expansion of visceral adipose tissue by lipolysis, and participate in the cachectic state of the advanced stages of HF. They are involved in lipid metabolism by promoting mitochondrial biogenesis and adipocyte energy expenditure via the p38 MAPK pathway. Further, it has been proposed that NPs may also play a role in the pathogenesis of the metabolic syndrome where a lower than expected circulating NP concentration is observed [58,59]. Interestingly, the strength of the relationships between NP (MR-proANP) and body mass index, waist circumference, and diastolic blood pressure were significantly stronger among the young adults compared with the adolescents [60]. These lower circulating concentrations of NPs could play a role in the early stages of hypertension development.

## 3. Pathophysiology of Cardiac Natriuretic Peptides

### 3.1. Mechanisms Stimulating the Cardiac Natriuretic System in Pathological Situations

#### 3.1.1. Hemodynamics

In the early stages of HF, the sympathetic nervous system (SNS) and the RAAS response play a compensatory role, supporting cardiac output and increasing peripheral vasoconstriction in order to maintain circulatory homeostasis. However, prolonged activation of both systems becomes detrimental and contributes to the progression and aggravation of HF, ultimately leading to congestion. In addition to the classical components of neuroendocrine activation, other regulatory systems are involved, namely, kinins, NPs, endothelin, erythropoietin, prostaglandins, and adrenomedullin [61,62]. If the compensatory activation of the SNS and RAAS becomes harmful over time and ultimately affects the prognosis, the activation of kinins and the NP system plays a favorable and protective role in HF. The increase in the production of ANP and BNP by the heart in response to myocardial stretch due to the hemodynamic variations is related not only to the stimulation of gene expression (especially by the p38MAPK/NF-ƘB pathway) but also to the activation of ion channels sensitive to mechanical stimuli [63,64].

The mechanisms of cardiac response are different depending on the duration of acute (in hours) or chronic (greater than 1 week) hemodynamic variation [65]. Acute atrial stretching is a mechanism of stretch–secretion coupling from a pool of NPs previously synthesized and stored in atrial cardiomyocytes, thus allowing secretion in bursts [66]. The chronic hemodynamic overload observed, particularly in cases of HF, stimulates the synthesis and secretion of ANP and BNP in cardiomyocytes not only atrial but also ventricular, by reactivating the fetal genetic program [67].

Interestingly, Gulati et al. [68] observed in the PRADA study that metoprolol, a β-adrenergic antagonist therapy, was associated with higher concentrations of BNP and NT-proBNP. One potential mechanism is the reduction in heart rate which induced higher end-diastolic volume and therefore an increased cardiomyocyte stretch.

#### 3.1.2. Heart Transplantation

##### Chronic Inflammation

It seems that diastolic dysfunction, commonly accepted as the main cause of an increase in circulating BNP [69], is not always the *primum movens*. In heart transplant patients, TNF-α (tumor necrosis factor-α) levels are chronically elevated in the graft in the absence of any obvious histopathological or clinical rejection episodes and in the presence of normal left ventricular function [70]. In vitro, TNF-α and IL-1β (interleukin-1β) selectively enhance BNP gene transcription via the p38 MAPK signaling pathway [71]. Lipopolysaccharide (LPS), an inflammation mediator, is also able to directly stimulate BNP gene expression in rats by specifically targeting the GATA enhancer located in the proximal part of the BNP promoter. In humans, other cytokines such as RANTES (Regulated on activation, normal T expressed and secreted), NAP-2 (neutrophil-activating protein-2), and IGFBP-1 (insulin growth factor binding protein-1) present in biopsies of rejected heart transplants, are selectively correlated to patients’ BNP plasma and not to plasma ANP [72]. This inflammatory process therefore seems to be linked to a specific regulation of BNP dissociated from that of ANP and, therefore, highlights that BNP regulation can be dissociated from any hemodynamic variation [73].

##### Chronic Hypoxia

Ventricular hypertrophy often occurs after heart transplantation, and Gramley et al. [74] report a progressive increase in myocardial fibrosis from 12.6% ± 6.5% in the first post-transplant period to 28.8% ± 7.8% at 10 years of transplantation. Several causal factors are mentioned in the genesis of this graft fibrosis: ciclosporin A treatment, ischemic time between harvesting and transplantation of the graft, and graft vasculopathy. This cardiac fibrosis would increase the distance of oxygen diffusion from capillaries to cardiomyocytes by accumulating collagen in the interstitium around the cardiomyocytes. This would promote acute/chronic local hypoxia that would trigger cellular adaptation to these conditions. Immunohistochemical analysis of hypoxia-induced myocardial protein expression showed an increase in early and late expression of factor 1α (HIF1α), a progressive increase in prolyl hydroxylase 3 (PHD3) and vascular endothelial growth factor (VEGF). Interestingly, hypoxia-sensitive elements were found in the BNP and ANP gene promoter sequences [75,76]. In relation to this process, Stockmann et al. [77] studied the effects of oxygenation on enlarged cardiac ventricles and showed in rats that, when normoxia conditions are restored, the ANP content decreases towards control rat levels despite persistent hypertrophy. Arjamaa suggests that the role of NPs in hypoxia conditions is probably not to counterbalance pressure changes in the circulation, but to regulate oxygen transport causing a contraction of blood volume (diuresis, natriuresis, vascular permeability) leading to hemoconcentration and increased oxygen transport capacity per unit volume of blood [78]. In addition, Anttila et al. [79] showed in a Langendorff rat beating-heart device that the BNP level of the infusate increases when the oxygen pressure of the infused solution decreases. The effect of oxygen was independent of the degree of mechanical stretching of the myocardium, even after the heart rate decreased while the pressure conditions remained constant. From a therapeutic perspective of muscle ischemia, it is interesting to note that pre-treatment of recombinant BNP prevented cardiac and skeletal muscle damage under ischemia-reperfusion conditions [52,57].

#### 3.1.3. Neuroendocrine Factors

Several other neuroendocrine factors can modulate cardiac NPs’ secretion by targeting a GATA enhancer; adrenergic agonists, endothelin-1, glucocorticoids, acetylcholine, prostaglandins, thyroid hormones, and angiotensin II can activate the production of ANP and BNP [16]. The involvement of glucagon-like peptide-1 in regulating the cardiac secretion of ANP and reducing blood pressure has been highlighted by Kim [80].

### 3.2. Mechanisms of Resistance to Natriuretic Peptides (Figure 2)

#### 3.2.1. Renal Resistance

Natriuretic peptides play an important role in chronic HF, protecting the patient from hydro-sodium overload and, thus, delaying progression to cardiac decompensation. Nevertheless, despite their increasing secretion during disease evolution, these NPs gradually lose their natriuretic effect and a fall in natriuresis is observed, suggesting the appearance of renal resistance to these NPs. In animals with HF, a decrease in urinary cGMP concentration is observed [81]. Several hypotheses concerning the mechanisms of renal resistance to NP have been put forward: a local increase in NP degradation, dephosphorylation of NPR-A [82,83,84], and/or a decrease in renal concentration of NPR-A [85]. Nevertheless, an alteration of the intracellular signaling pathway of NPR-A, an increase in the activity of PDEs or a predominant action of anti-natriuretic and vasoconstrictor systems such as RAAS (inducing “desensitization” of receptors and opposing the tubular activity of ANP), the sympathetic system, endothelin, and/or arginine vasopressin also seem to play an important role [86].

#### 3.2.2. The BNP Paradox

Beyond mechanisms of renal resistance to NPs, the BNP paradox in HF is defined as alterations in physiological responses (e.g., increased vasoconstriction, decreases in diuresis, natriuresis, urinary cGMP excretion) resulting in clinical aggravation, despite a significant increase in BNP plasma levels using standard immunoreactive assay kits [87]. This phenomenon might be linked to the presence of altered and biologically inactive circulating molecular forms of BNP, which are not distinguishable from biologically active BNP1-32 by the assay kits marketed nowadays [88]. Indeed, standard marketed BNP and NT-proBNP assay kits might not be able to differentiate between the circulating forms of BNP, NT-proBNP and proBNP. Further, kits also overestimate the BNP1-32 levels, because they also recognize less active BNP forms. Accordingly, Liang et al. [89] using Western blot analysis observed in the plasma of HF patients the presence of circulating plasma forms of BNP of low and high molecular weights, the latter being the most frequent and producing 6 to 8 times less cGMP in endothelial cells than BNP.

A BNP precursor, pre-proBNP (134 aa), is stored in the cardiomyocyte and secreted almost exclusively by ventricular myocytes. It splits into a pro-hormone called pro-BNP (108 aa) and the signal peptide (26 aa). In response to the stretching of the cardiomyocyte secondary to an increase in intra-cardiac pressure and/or volume overload, proBNP1-108 would remain classically in the intracellular compartment where it would be divided into two fragments released into the blood: BNP 32 aa (active form) and NT-proBNP 76 aa (inactive form). However, studies have shown more recently that proBNP1-108, although synthesized in atrial and ventricular myocytes, is also present in plasma before being separated in NT-proBNP1-76 and BNP1-32 active by the two natriuretic convertase propeptides: corin and furin [90,91,92,93].

The different forms of low molecular weight BNP are related to the presence of BNP1-32, mainly degraded to BNP3-32 or to BNP8-32 by DPP IV [94,95], and to BNP5-32 by neutral endopeptidase (neprilysin) [96]. Rather than BNP1-32, BNP3-32 may be the predominant form of BNP. In addition, proBNP1-108 can also be proteolyzed by DPP IV as circulating proBNP3-108, as well as NT-proBNP in essentially truncated circulating forms [89,97,98]. By mass spectral analysis, some authors observed a rapid degradation of BNP1-32 with the presence of different degraded forms in the plasma of HF patients. This spectral analysis detected not only a low level of BNP1-32 but also the presence of degraded and/or no functional forms of BNP, while the plasma level of BNP determined by the Triage Biosite^®^ method was very high in the same patient samples, suggesting an acceleration of BNP degradation in HF. The presence of altered BNP and circulating proBNP1-108 is predominant, at higher levels than in normal subjects [89,99,100]. Moreover, proBNP1-108 would even be the major immunoreactive form of BNP in HF patients, whereas it appears to be 6 to 8 times less active than BNP [101]. 

Several explanations of this heterogeneity of circulating forms of BNP in HF are put forward. The proNP convertases corin and furin, by their action of converting proBNP1-108 into active BNP, could play an important role in HF conditions. Semenov et al. reported that the corin transformation process of proBNP1-108 into active BNP could be suppressed by glycosylation of proBNP1-108 near the cleavage site of this enzyme. Thus, O-glycosylation could play a key role in inhibiting the cleavage process of proBNP1-108 by pro-peptide convertases [102]. About 70% of proBNP molecules circulating in HF patients are glycosylated, making the molecule resistant to the proteolytic action of corin and furin to produce BNP and NT-proBNP [103,104]. Considering that the circulating form of proBNP1-108 can be predominantly glycosylated and, thus, become resistant to the corin action, this would imply that the glycosylation state may be a key point to be taken into account, as it could lead to an underestimation of the circulation levels of BNP, NT-proBNP1-76 or proBNP1-108 (for review, see References [93,105]). Moreover, the serum of HF patients involves a delayed conversion process from proBNP to mature BNP, probably secondary to reduced levels of soluble corin [92]. 

In addition, the activity of DPP IV is increased in the serum of HF patients, and not only promotes the degradation of BNP 1-32 into degraded and biologically inactive circulating forms, but also increases the transformation of proBNP1-108 into a truncated form 3-108 [95,105,106]. Interestingly, inhibition of DPP IV would improve heart and kidney functions in a porcine HF model [107].

In summary, not only the HF patients would be deficient in biologically active BNP, but the currently available BNP and NT-proBNP assay kits do not effectively distinguish between circulating forms of BNP, NT-proBNP, and proBNP1-108. 

This lack of specificity of BNP assay kits described in HF might partly explain the persistent high plasma levels of BNP in the heart transplant population despite a recovery in hemodynamic conditions [108]. Moreover, even if ESC and ACC/AHA guidelines confirm the clinical utility of serum BNP or NT-proBNP for establishing disease and prognostic in chronic and acute HF, biomarker-“guided” therapy has produced inconsistent results in randomized controlled trials for the purpose of reducing hospitalizations or mortality [109]. In addition to NPs, other plasmatic biomarkers dosages as GDF-15 (growth differentiation factor 15) in ischemic HF, a heart-derived hormone that regulates pediatric body growth and would coordinate cardiac function with NPs [110], might be an interesting complementary tool in HF diagnosis and clinical follow-up [111]. 

### 3.3. Involvement of PDEs in Cardiac Pathologies

**Figure 3:**
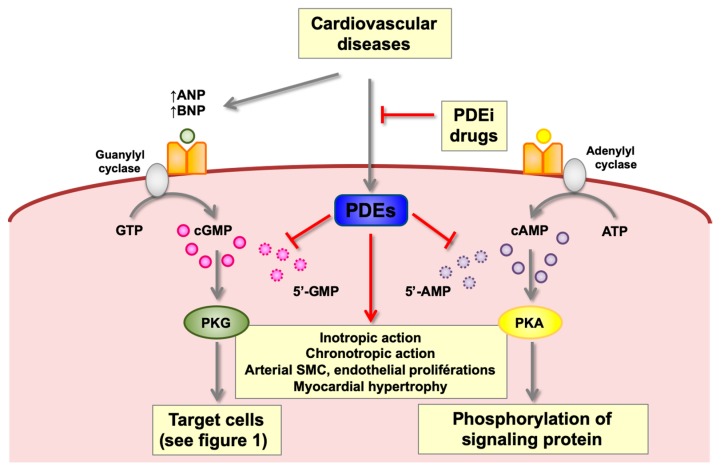
Cyclic nucleotide phosphodiesterases family implications and therapeutic targets in cardiovascular diseases. Cardiovascular diseases alter specific PDE(s) either by increasing or decreasing PDE activity in a compartmentalized manner in conjunction with receptor responses. Not only might the cGMP pathway be impacted by natriuretic peptides, but also the cAMP pathway. Since PDE2 and PDE3, which are oppositely modulated by cGMP level, are both present in cardiomyocytes, dependently of their relative activities, they might also participate in the regulation of cardiac cGMP (see Figure 2). In that way, the alterations in natriuretic peptides might impact not only cGMP signaling, but also cAMP signaling. Thus, by overcoming specific characterized PDE alterations, it should be possible to restore a physiologic status by conceiving new PDE modulators, notably PDE inhibitors (PDEi). 5′ AMP, 5′ adenosine monophosphate; cAMP, adenosine 3′,5′-cyclic monophosphate; ANP, atrial natriuretic peptide; ATP, adenosine triphosphate; BNP, brain natriuretic peptide; 5′ GMP, 5′ guanosine monophosphate; cGMP, guanosine 3′,5′-cyclic monophosphate; GTP, guanosine triphosphate; PDE, cyclic nucleotide phosphodiesterase; PDEi, PDE inhibitors; PKA, protein kinase A; PKG, protein kinase G; SMC, smooth muscle cell.

A typical characteristic of cardiac failure is abnormal second messenger signaling due to the impaired synthesis and catabolism of cAMP and cGMP. A renewed interest has emerged recently in the literature on the participation of NPs [112] and PDEs [113] in different cardiac diseases, since actors which modulate the intracellular rate of cGMP might play a key role. Particularly, research on HF has shown that PDEs could represent new therapeutic targets. PDEs 1–5, 8, and 9 are dysregulated in HF, but only preclinical studies have established a role in cardiac regulation for all of these PDEs, while clinical data related to heart disease only exists for PDE3 and PDE5. Nevertheless, no inhibitors of PDEs are actually approved for chronic HF, neither with a reduced ejection fraction nor with a preserved ejection fraction (Figure 3).

PDE3 inhibitors, such as milrinone, have been characterized for the first time as new non-glycosidic and non-sympathomimetic cardiotonic agents capable of increasing the contractile strength of the myocardium [31]. Currently, milrinone use is limited to short-term treatment of acute congestive HF in intensive care, due to the induction of some unexplained deaths during chronic treatment [114]. Further, milrinone can induce arrhythmias and also inhibits PDE4 and PDE5 on the one hand, and acts at the same concentrations on the PDE3 of the sinus node responsible for the heartbeat, on the other hand [26].

Characterization of PDE3A and PDE3B and study of KO mouse models allow for focus on PDE3A, mainly expressed at the heart level and responsible for inotropic and chronotropic functions. NPs, by increasing intracellular cGMP, inhibit PDE3 [112] and, consequently, increase cAMP. However, their actions on the NPR-C oppose the synthesis of cAMP. Thus, below the NP receptors, cyclic nucleotide-dependent intracellular signaling regulated by PDEs is involved in a complex way in cellular responses, depending on pathophysiological alterations. 

Interestingly, the inhibition of cardiac PDE4 by rolipram potentiates the positive inotropic effect induced by forskolin or a PDE3 inhibitor, characterizing the involvement of PDE4 in the regulation of cardiac contraction [115].

In addition to the involvement of PDE3 in cardiac hypertrophy, a decrease in cAMP hydrolysis was shown during rat-induced HF by thoracic aorta ligation (5 weeks). This was associated with decreases in the expression of PDE3A, PDE4A, and PDE4B, which reduces the β-adrenergic signal [116]. When angiotensin II induces HF in rats (2 weeks), there is an increase in PDE 4 activity associated with an increase in PDE 4A expression, with no change in PDE3 [117].

In human atrial myocyte, it has been shown that increased PDE4 activity may be associated with a protection against atrial fibrillation [118]. Studies in the right ventricle of the enlarged human heart have shown overexpression of PDE5 and improved cardiac contraction with sildenafil, which is related to inhibition of PDE3 by cGMP [119]. In accordance with this work, an increase in the activity and expression (mRNA and protein) of PDE5 in angiotensin II-induced HF in rats is also observed [117]. A recent study conducted on ventricular cardiomyocytes from enlarged HF human hearts showed the presence and activity of PDE1, PDE3, PDE5 and variability of PDE4 in the failing human heart, while PDE2 and PDE7 are present, without being able to determine their enzymatic activities.

In the rat ventricle following an aortic ligation, PDE2 is over-regulated [120]. It has recently been shown that the activity and expression of PDE2 are increased in HF in humans and rats, and would have a cardio-protective effect by decreasing the adrenergic response [121], while inhibition of PDE2 would be anti-hypertrophic [122]. This apparent contradiction has been noted and may result from different techniques for overexpression of PDE2, leading the authors to suggest the use of PDE2 activator to resolve this contradiction [123].

There is an increase in the expression of PDE1A in the aorta associated with the development of tolerance to nitrated derivatives [124], while PDE1 promotes the proliferation of arterial smooth muscle [125]. In rat heart, during angiotensin II-induced HF, there is an increase in the hydrolytic activity of PDE1 with respect to cGMP (+130%), mRNA of PDE1A (+140%), and PDE1C (+54%) and protein expression of PDE1A (+30%) and PDE1C (+32%; +41%) associated with an increase in BNP mRNA expression (+254%) [109]. A study shows that mRNAs of PDE1A and PDE1C are also present in the human heart and that PDE1A plays a critical role in PKG-dependent cardiac hypertrophy, while the use of a PDE1 inhibitor would decrease protein synthesis [126]. 

All these data highlight the role of PDE regulation in the HF, and when NPs can no longer effectively regulate cGMP levels at the heart level, it is possible to act below them by modulating the activity of altered PDEs during the pathology.

## 4. Conclusions

The heart has an endocrine function that is ensured, among others, by cardiac NPs. Through their natriuretic and vasodilator effects, these cardiac NPs play a fundamental role in hydro-sodium regulation. Modulating blood volume, they help to regulate the blood pressure over the long term.

On the physio-pathological level, the increase in the production of ANP and BNP by the heart is stimulated not only by myocardial stretching secondary to hemodynamic variations but also by inflammation with the particularity that it specifically stimulates BNP synthesis, underlining a mode of BNP regulation that can be dissociated from any hemodynamic variation.

During HF, stimulation of the cardiac NP system is firstly beneficial. But, thereafter, its efficacy decreases leading to the “BNP paradox”, which resides in the fact that despite a significant increase in BNP plasma levels, as demonstrated by standard immunoreactive assay kits, the HF patients would ultimately be deficient in biologically active BNP.

In these pathological conditions, where the production of ANP and BNP is no longer effective in normalizing cardiac function, it is possible to act downstream by modulating the activity of PDEs, intervening in the intracellular signaling cascade, and taking over the control of the phosphorylation cascade. Differential control over cAMP and cGMP signaling in cardiomyocytes, provides potential therapeutic opportunities to counter HF. 

## Figures and Tables

**Table 1:** Cyclic nucleotide phosphodiesterases family.

Cardiac PDEs Family	Localization	Substrate	Action	Inhibition	References
**PDE1**	Heart, vascular SMC	1A and B: cGMP 1C: cAMP/cGMP	Regulation of cyclic nucleotides and calcium		[26,27,28]
**PDE2**	Heart, endothelial cells	cAMP/cGMP	Feedback of basal cGMP and cAMP in response to an increase in cGMP (production of NO, ANP and BNP)		[26,27,29]
**PDE3**	Heart, endothelial cells, vascular SMC	cGMP > cAMP	Interaction between the regulatory pathways of cAMP and cGMP	Increases cardiac strength while inducing vascular relaxation	[26,30,31]
**PDE4**	Heart, vascular SMC, endothelial cells and immunocytes	cAMP	Insensitivity to cGMP differentiates from PDE3		[32]
**PDE5**	Heart, vascular SMC and endothelium	cGMP		Treatment of erectile dysfunction, beneficial effect in HF, treatment of PAH	[33,34,35,36,37]
**PDE8**	Cardiomyocytes in the ventricle	cAMP	Control of cardiac function at the excitation-contraction coupling		[38]
**PDE9**	Dilated cardiomyopathies	cGMP			[39]

cAMP, adenosine 3′,5′-cyclic monophosphate; cGMP, guanosine 3′,5′-cyclic monophosphate; HF, heart failure; PAH, pulmonary artery hypertension; PDEs, cyclic nucleotide phosphodiesterases; SMC, smooth muscle cell.

## References

[1] Dussaule J.-C., Ledoux S., Pham I., Hittinger L., Berthezène F., Castaigne A., Dubois-Randé J.-L., Plouin P.F. (1998). Système endocrinien cardiaque, coeur et vaisseaux. Hormones, Coeur et Vaisseaux.

[2] Talha S., Piquard F., Geny B. (2015). Fonction endocrine du cœur. EMC Cardiol..

[3] Chiba A., Watanabe-Takano H., Miyazaki T., Mochizuki N. (2018). Cardiomyokines from the heart. Cell. Mol. Life Sci..

[4] Ciccarelli M., Sorriento D., Coscioni E., Iaccarino G., Santulli G., Schisler J.C., Lang C.H., Willis M.S. (2017). Adrenergic Receptors. Endocrinology of the Heart in Health and Disease: Integrated, Cellular, and Molecular Endocrinology of the Heart (Chapter: 11).

[5] de Bold A.J., Borenstein H.B., Veress A.T., Sonnenberg H. (1981). A rapid and potent natriuretic response to intravenous injection of atrial myocardial extract in rats. Life Sci..

[6] Sudoh T., Kangawa K., Minamino N., Matsuo H. (1988). A new natriuretic peptide in porcine brain. Nature.

[7] Moyes A.J., Hobbs A.J. (2019). C-type Natriuretic Peptide: A Multifaceted Paracrine Regulator in the Heart and Vasculature. Int. J. Mol. Sci..

[8] Meyer M., Richter R., Forssmann W.G. (1998). Urodilatin, a natriuretic peptide with clinical implications. Eur. J. Med. Res..

[9] Nakamura S., Naruse M., Naruse K., Kawana M., Nishikawa T., Hosoda S., Tanaka I., Yoshimi T., Yoshihara I., Inagami T. (1991). Atrial natriuretic peptide and brain natriuretic peptide coexist in the secretory granules of human cardiac myocytes. Am. J. Hypertens..

[10] Durocher D., Grepin C., Nemer M. (1998). Regulation of gene expression in the endocrine heart. Recent. Prog. Horm. Res..

[11] Fu S., Ping P., Wang F., Luo L. (2018). Synthesis, secretion, function, metabolism and application of natriuretic peptides in heart failure. J. Biol. Eng..

[12] Edwards B.S., Zimmerman R.S., Schwab T.R., Heublein D.M., Burnett J.C. (1988). Atrial stretch, not pressure, is the principal determinant controlling the acute release of atrial natriuretic factor. Circ. Res..

[13] Stoupakis G., Klapholz M. (2003). Natriuretic peptides: Biochemistry, physiology, and therapeutic role in heart failure. Heart Dis..

[14] Potter L.R., Abbey-Hosch S., Dickey D.M. (2006). Natriuretic peptides, their receptors, and cyclic guanosine monophosphate-dependent signaling functions. Endocr. Rev..

[15] Cohen D., Koh G.Y., Nikonova L.N., Porter J.G., Maack T. (1996). Molecular determinants of the clearance function of type C receptors of natriuretic peptides. J. Biol. Chem..

[16] Ralat L.A., Guo Q., Ren M., Funke T., Dickey D.M., Potter L.R., Tang W.J. (2011). Insulin-degrading enzyme modulates the natriuretic peptide-mediated signaling response. J. Biol. Chem..

[17] Gupta D.K., Wang T.J. (2015). Natriuretic Peptides and Cardiometabolic Health. Circ. J..

[18] De Bold A.J., Bruneau B.G., Kuroski de Bold M.L. (1996). Mechanical and neuroendocrine regulation of the endocrine heart. Cardiovasc. Res..

[19] Keravis T., Lugnier C. (2012). Cyclic nucleotide phosphodiesterase (PDE) isozymes as targets of the intracellular signalling network: Benefits of PDE inhibitors in various diseases and perspectives for future therapeutic developments. Br. J. Pharmacol..

[20] Lincoln T.M., Dey N., Sellak H. (2001). Invited review: cGMP-dependent protein kinase signaling mechanisms in smooth muscle: From the regulation of tone to gene expression. J. Appl. Physiol..

[21] Vaandrager A.B., Hogema B.M., de Jonge H.R. (2005). Molecular properties and biological functions of cGMP-dependent protein kinase II. Front. Biosci..

[22] Rubattu S., Sciarretta S., Morriello A., Calvieri C., Battistoni A., Volpe M. (2010). NPR-C: A component of the natriuretic peptide family with implications in human diseases. J. Mol. Med. (Berl.).

[23] Azevedo M.F., Faucz F.R., Bimpaki E., Horvath A., Levy I., de Alexandre R.B., Ahmad F., Manganiello V., Stratakis C.A. (2014). Clinical and molecular genetics of the phosphodiesterases (PDEs). Endocr. Rev..

[24] Maurice D.H., Ke H., Ahmad F., Wang Y., Chung J., Manganiello V.C. (2014). Advances in targeting cyclic nucleotide phosphodiesterases. Nat. Rev. Drug. Discov..

[25] Ahmad F., Murata T., Shimizu K., Degerman E., Maurice D., Manganiello V. (2015). Cyclic nucleotide phosphodiesterases: Important signaling modulators and therapeutic targets. Oral Dis..

[26] Komas N., Lugnier C., Le Bec A., Serradeil-Le Gal C., Barthélémy G., Stoclet J.-C. (1989). Differential sensitivity to cardiotonic drugs of cyclic AMP phosphodiesterases isolated from canine ventricular and sinoatrial-enriched tissues. J. Cardiovasc. Pharmacol..

[27] Lugnier C., Schoeffter P., Le Bec A., Strouthou E., Stoclet J.-C. (1986). Selective inhibition of cyclic nucleotide phosphodiesterases of human, bovine and rat aorta. Biochem. Pharmacol..

[28] Stoclet J.-C., Boulanger-Saunier C., Lassegue B., Lugnier C. (1988). Cyclic nucleotides and calcium regulation in heart and smooth muscle cells. Ann. N. Y. Acad. Sci..

[29] Lugnier C., Schini V.B. (1990). Characterization of cyclic nucleotide from cultured bovine aortic endothelial cells. Biochem. Pharmacol..

[30] Komas N., Lugnier C., Stoclet J.-C. (1991). Endothelium-dependent and independent relaxation of the rat aorta by cyclic nucleotide phosphodiesterase inhibitors. Br. J. Pharmacol..

[31] Bristol J.A., Sircar I., Moos W.H., Evans D.B., Weishaar R.E. (1984). Cardiotonic agents: 1. 4,5-Dihydro-6-[4-(1H-imidazol-1-yl)phenyl]-3 (2H)-pyridazinones: Novel positive inotropic agents for the treatment of congestive heart failure. J. Med. Chem..

[32] Lugnier C., Stierlé A., Beretz A., Schoeffter P., Lebec A., Wermuth C.G., Cazenave J.-P., Stoclet J.-C. (1983). Tissue and substrate specificity of inhibition by alkoxy-aryl-lactams of platelet and arterial smooth muscle cyclic nucleotide phosphodiesterases relationship to pharmacological activity. Biochem. Biophys. Res. Commun..

[33] Boolell M., Allen M.J., Ballard S.A., Gepi-Attee S., Muirhead G.J., Naylor A.M., Osterloh I.H., Gingell C. (1996). Sildenafil: An orally active type 5 cyclic GMP-specific phosphodiesterase inhibitor for the treatment of penile erectile dysfunction. Int. J. Impot. Res..

[34] Yafi F.A., Sharlip I.D., Becher E.F. (2018). Update on the safety of phosphodiesterase type 5 inhibitors for the treatment of erectile dysfunction. Sex. Med. Rev..

[35] Takimoto E., Champion H.C., Belardi D., Moslehi J., Mongillo M., Mergia E., Montrose D.C., Isoda T., Aufiero K., Zaccolo M. (2005). cGMP catabolism by phosphodiesterase 5A regulates cardiac adrenergic stimulation by NOS3-dependent mechanism. Circ. Res..

[36] Ghazi M., Vicenzi M., Arena R., Guazzi M.D. (2011). PDE5 inhibition with sildenafil improves left ventricular diastolic function, cardiac geometry, and clinical status in patients with stable systolic heart failure: Results of a 1-year, prospective, randomized, placebo-controlled study. Circ. Heart Fail..

[37] Klinger J.R., Thaker S., Houtchens J., Preston I.R., Hill N.S., Farber H.W. (2006). Pulmonary hemodynamic responses to brain natriuretic peptide and sildenafil in patients with pulmonary arterial hypertension. Chest.

[38] Patrucco E., Albergine M.S., Santana L.F., Beavo J.A. (2010). Phosphodiesterase 8A (PDE8A) regulates excitation-contraction coupling in ventricular myocytes. J. Mol. Cell. Cardiol..

[39] Lee D.I., Zhu G., Sasaki T., Cho G.S., Hamdani N., Holewinski R., Jo S.H., Danner T., Zhang M., Rainer P.P. (2015). Phosphodiesterase 9A controls nitric-oxide independent cGMP and hypertrophic heart disease. Nature.

[40] Theilig F., Wu Q. (2015). ANP-induced signaling cascade and its implications in renal pathophysiology. Am. J. Physiol. Renal. Physiol..

[41] Maack T. (1996). Role of atrial natriuretic factor in volume control. Kidney Int..

[42] Bae E.H., Ma S.K., Lee J., Kim S.W. (2011). Altered regulation of renal nitric oxide and atrial natriuretic peptide systems in angiotensin II-induced hypertension. Regul. Pept..

[43] Chen H.H., Burnett J.C. (2000). Natriuretic peptides in the pathophysiology of congestive heart failure. Curr. Cardiol. Rep..

[44] McGrath M.F., de Bold M.L., de Bold A.J. (2005). The endocrine function of the heart. Trends Endocrinol. Metab..

[45] Tarjan E., Denton D.A., Weisinger R.S. (1988). Atrial natriuretic peptide inhibits water and sodium intake in rabbits. Regul. Pept..

[46] Matsukawa T., Miyamoto T. (2011). Angiotensin II-stimulated secretion of arginine vasopressin is inhibited by atrial natriuretic peptide in humans. Am. J. Physiol. Regul. Integr. Comp. Physiol..

[47] Itoh H., Pratt R.E., Dzau V.J. (1991). Interaction of atrial natriuretic polypeptide and angiotensin II on protooncogene expression and vascular cell growth. Biochem. Biophys. Res. Commun..

[48] Tamura N., Ogawa Y., Chusho H., Nakamura K., Nakao K., Suda M., Kasahara M., Hashimoto R., Katsuura G., Mukoyama M. (2000). Cardiac fibrosis in mice lacking brain natriuretic peptide. Proc. Natl. Acad. Sci. USA.

[49] Das B.B., Raj S., Solinger R. (2009). Natriuretic peptides in cardiovascular diseases of fetus, infants and children. Cardiovasc. Hematol. Agents Med. Chem..

[50] Schwachtgen L., Herrmann M., Georg T., Schwarz P., Marx N., Lindinger A. (2005). Reference values of NT-proBNP serum concentrations in the umbilical cord blood and in healthy neonates and children. Z. Kardiol..

[51] Becker J.R., Chatterjee S., Robinson T.Y., Bennett J.S., Panáková D., Galindo C.L., Zhong L., Shin J.T., Coy S.M., Kelly A.E. (2014). Differential activation of natriuretic peptide receptors modulates cardiomyocyte proliferation during development. Development.

[52] D’Souza S.P., Yellon D.M., Martin C., Schulz R., Heusch G., Onody A., Ferdinandy P., Baxter G.F. (2003). B-type natriuretic peptide limits infarct size in rat isolated hearts via KATP channel opening. Am. J. Physiol. Heart Circ. Physiol..

[53] Fiscus R.R., Tu A.W., Chew S.B. (2001). Natriuretic peptides inhibit apoptosis and prolong the survival of serum-deprived PC12 cells. Neuroreport.

[54] Sun Y., Zhang Y., Yan M., Wu Y., Zheng X. (2009). B-type natriuretic peptide-induced cardioprotection against reperfusion is associated with attenuation of mitochondrial permeability transition. Biol. Pharm. Bull..

[55] Wang T.N., Ge Y.K., Li J.Y., Zeng X.H., Zheng X.X. (2007). B-type natriuretic peptide enhances mild hypoxia-induced apoptotic cell death in cardiomyocytes. Biol. Pharm. Bull..

[56] De Vito P., Incerpi S., Pedersen J.Z., Luly P. (2010). Atrial natriuretic peptide and oxidative stress. Peptides.

[57] Talha S., Bouitbir J., Charles A.-L., Zoll J., Goette-Di Marco P., Meziani F., Piquard F., Geny B. (2013). Pretreatment with brain natriuretic peptide reduces skeletal muscle mitochondrial dysfunction and oxidative stress after ischemia-reperfusion. J. Appl. Physiol..

[58] Schlueter N., de Sterke A., Willmes D.M., Spranger J., Jordan J., Birkenfeld A.L. (2014). Metabolic actions of natriuretic peptides and therapeutic potential in the metabolic syndrome. Pharmacol. Ther..

[59] Sengenès C., Berlan M., De Glisezinski I., Lafontan M., Galitzky J. (2000). Natriuretic peptides: A new lipolytic pathway in human adipocytes. FASEB J..

[60] Goharian T.S., Goetze J.P., Faber J., Andersen L.B., Grøntved A., Jeppesen J.L. (2017). Associations of proatrial natriuretic peptide with components of the metabolic syndrome in adolescents and young adults from the general population. Am. J. Hypertens..

[61] Magri P., Rao M.A., Cangianiello S., Bellizzi V., Russo R., Mele A.F., Andreucci M., Memoli B., De Nicola L., Volpe M. (1988). Early impairment of renal hemodynamic reserve in patients with asymptomatic heart failure is restored by angiotensin II antagonism. Circulation.

[62] Mastromarino V., Volpe M., Musumeci M.B., Autore C., Conti E. (2011). Erythropoietin and the heart: Facts and perspectives. Clin. Sci..

[63] Liang F., Gardner D.G. (1999). Mechanical strain activates BNP gene transcription through a p38/NF-κB—dependent mechanism. J. Clin. Investig..

[64] Sadoshima J., Jahn L., Takahashi T., Kulik T.J., Izumo S. (1992). Molecular characterization of the stretch-induced adaptation of cultured cardiac cells. An in vitro model of load-induced cardiac hypertrophy. J. Biol. Chem..

[65] Ogawa T., de Bold A.J. (2014). The heart as an endocrine organ. Endocr. Connect..

[66] Kuroski de Bold M.L., de Bold A.J. (1991). Stretch-secretion coupling in atrial cardiocytes. Dissociation between atrial natriuretic factor release and mechanical activity. Hypertension.

[67] Yokota N., Bruneau B.G., Fernandez B.E., de Bold M.L., Piazza L.A., Eid H., de Bold A.J. (1995). Dissociation of cardiac hypertrophy, myosin heavy chain isoform expression, and natriuretic peptide production in DOCA-salt rats. Am. J. Hypertens..

[68] Gulati G., Heck S.L., Røsjø H., Ree A.H., Hoffmann P., Hagve T.A., Norseth J., Gravdehaug B., Steine K., Geisler J. (2017). Neurohormonal blockade and circulating cardiovascular biomarkers during anthracycline therapy in breast cancer patients: Results from the PRADA (Prevention of Cardiac Dysfunction During Adjuvant Breast Cancer Therapy) study. J. Am. Heart Assoc..

[69] Geny B., Charloux A., Lampert E., Lonsdorfer J., Haberey P., Piquard F. (1998). Enhanced brain natriuretic peptide response to peak exercise in heart transplant recipients. J. Appl. Physiol..

[70] Torre-Amione G., MacLellan W., Kapadia S., Weilbaecher D., Farmer J., Young J., Mann D. (1998). Tumor necrosis factor-alpha is persistently expressed in cardiac allografts in the absence of histological or clinical evidence of rejection. Transplant. Proc..

[71] Ma K.K., Ogawa T., De Bold A.J. (2004). Selective upregulation of cardiac brain natriuretic peptide at the transcriptional and translational levels by pro-inflammatory cytokines and by conditioned medium derived from mixed lymphocyte reactions via p38 MAP kinase. J. Mol. Cell. Cardiol..

[72] Vesely D.L., de Bold A.J. (2009). Cardiac natriuretic peptides gene expression and secretion in inflammation. J. Investig. Med..

[73] Talha S., Charloux A., Enache I., Piquard F., Geny B. (2011). Mechanisms involved in increased plasma brain natriuretic peptide after heart transplantation. Cardiovasc. Res..

[74] Gramley F., Lorenzen J., Pezzella F., Kettering K., Himmrich E., Plumhans C., Koellensperger E., Munzel T. (2009). Hypoxia and myocardial remodeling in human cardiac allografts: A time-course study. J. Heart Lung Transplant..

[75] Chun Y.S., Hyun J.Y., Kwak Y.G., Kim I.S., Kim C.H., Choi E., Kim M.S., Park J.W. (2003). Hypoxic activation of the atrial natriuretic peptide gene promoter through direct and indirect actions of hypoxia-inducible factor-1. Biochem. J..

[76] Weidemann A., Klanke B., Wagner M., Volk T., Willam C., Wiesener M.S., Eckardt K.U., Warnecke C. (2008). Hypoxia, via stabilization of the hypoxia-inducible factor HIF-1alpha, is a direct and sufficient stimulus for brain-type natriuretic peptide induction. Biochem. J..

[77] Stockmann P.T., Will D.H., Sides S.D., Brunnert S.R., Wilner G.D., Leahy K.M., Wiegand R.C., Needleman P. (1988). Reversible induction of right ventricular atriopeptin synthesis in hypertrophy due to hypoxia. Circ. Res..

[78] Arjamaa O. (2014). Physiology of natriuretic peptides: The volume overload hypothesis revisited. World. J. Cardiol..

[79] Anttila K., Streng T., Pispa J., Vainio M., Nikinmaa M. (2017). Hypoxia exposure and B-type natriuretic peptide release from Langendorff heart of rats. Acta Physiol. (Oxf.).

[80] Kim M., Platt M.J., Shibasaki T., Quaggin S.E., Backx P.H., Seino S., Simpson J.A., Drucker D.J. (2013). GLP-1 receptor activation and Epac2 link atrial natriuretic peptide secretion to control of blood pressure. Nat. Med..

[81] Abassi Z., Burnett J.C., Grushka E., Hoffman A., Haramati A., Winaver J. (1991). Atrial natriuretic peptide and renal cGMP in rats with experimental heart failure. Am. J. Physiol. Regul. Integr. Comp. Physiol..

[82] Chen H.H., Schirger J.A., Chau W.L., Jougasaki M., Lisy O., Redfield M.M., Barclay P.T., Burnett J.C. (1999). Renal response to acute neutral endopeptidase inhibition in mild and severe experimental heart failure. Circulation.

[83] Margulies K.B., Burnett J.C. (1994). Inhibition of cyclic GMP phosphodiesterases augments renal responses to atrial natriuretic factor in congestive heart failure. J. Card. Fail..

[84] Supaporn T., Sandberg S.M., Borgeson D.D., Heublein D.M., Luchner A., Wei C.M., Dousa T.P., Burnett J.C. (1996). Blunted cGMP response to agonists and enhanced glomerular cyclic 3′, 5′-nucleotide phosphodiesterase activities in experimental congestive heart failure. Kidney Int..

[85] Bryan P.M., Xu X., Dickey D.M., Chen Y., Potter L.R. (2007). Renal hyporesponsiveness to atrial natriuretic peptide in congestive heart failure results from reduced atrial natriuretic peptide receptor concentrations. Am. J. Physiol. Renal Physiol..

[86] Charloux A., Piquard F., Doutreleau S., Brandenberger G., Geny B. (2003). Mechanisms of renal hyporesponsiveness to ANP in heart failure. Eur. J. Clin. Investig..

[87] Díez J. (2017). Chronic heart failure as a state of reduced effectiveness of the natriuretic peptide system: Implications for therapy. Eur. J. Heart Fail..

[88] Heublein D.M., Huntley B.K., Boerrigter G., Cataliotti A., Sandberg S.M., Redfield M.M., Burnett J.C. (2007). Immunoreactivity and guanosine 3′,5′-cyclic monophosphate activating actions of various molecular forms of human B-type natriuretic peptide. Hypertension.

[89] Lewis L.K., Raudsepp S.D., Yandle T.G., Prickett T.C., Richards A.M. (2017). Development of a BNP1-32 immunoassay that does not cross-react with proBNP. Clin. Chem..

[90] Yan W., Sheng N., Seto M., Morser J., Wu Q. (1999). Corin, a mosaic transmembrane serine protease encoded by a novel cDNA from human heart. J. Biol. Chem..

[91] Semenov A.G., Tamm N.N., Seferian K.R., Postnikov A.B., Karpova N.S., Serebryanaya D.V., Koshkina E.V., Krasnoselsky M.I., Katrukha A.G. (2010). Processing of pro-B-type natriuretic peptide: Furin and corin as candidate convertases. Clin. Chem..

[92] Huntley B.K., Sandberg S.M., Heublein D.M., Sangaralingham S.J., Burnett J.C., Ichiki T. (2015). Pro-B-type natriuretic peptide-1-108 processing and degradation in human heart failure. Circ. Heart Fail..

[93] Ichiki T., Huntley B.K., Burnett J.C. (2013). BNP molecular forms and processing by the cardiac serine protease corin. Adv. Clin. Chem..

[94] Brandt I., Lambeir A.M., Ketelslegers J.M., Vanderheyden M., Scharpé S., De Meester I. (2006). Dipeptidyl-peptidase IV converts intact B-type natriuretic peptide into its des-SerPro form. Clin. Chem..

[95] Volpe M., Carnovali M., Mastromarino V. (2016). The natriuretic peptides system in the pathophysiology of heart failure: From molecular basis to treatment. Clin. Sci. (Lond.).

[96] Pankow K., Wang Y., Gembardt F., Krause E., Sun X., Krause G., Schultheiss H.P., Siems W.E., Walther T. (2007). Successive action of meprin A and neprilysin catabolizes B-type natriuretic peptide. Circ. Res..

[97] Ala-Kopsala M., Magga J., Peuhkurinen K., Leipälä J., Ruskoaho H., Leppäluoto J., Vuolteenaho O. (2004). Molecular heterogeneity has a major impact on the measurement of circulating N-terminal fragments of A- and B-type natriuretic peptides. Clin. Chem..

[98] Suzuki T., Israr M.Z., Heaney L.M., Takaoka M., Squire I.B., Ng L.L. (2017). Prognostic role of molecular forms of B-Type natriuretic peptide in acute heart failure. Clin. Chem..

[99] Niederkofler E.E., Kiernan U.A., O’Rear J., Menon S., Saghir S., Protter A.A., Nelson R.W., Schellenberger U. (2008). Detection of endogenous B-type natriuretic peptide at very low concentrations in patients with heart failure. Circ. Heart Fail..

[100] Hawkridge A.M., Heublein D.M., Bergen H.R., Cataliotti A., Burnett J.C., Muddiman D.C. (2005). Quantitative mass spectral evidence for the absence of circulating brain natriuretic peptide (BNP-32) in severe human heart failure. Proc. Natl. Acad. Sci. USA.

[101] Seferian K.R., Tamm N.N., Semenov A.G., Mukharyamova K.S., Tolstaya A.A., Koshkina E.V., Kara A.N., Krasnoselsky M.I., Apple F.S., Esakova T.V. (2007). The brain natriuretic peptide (BNP) precursor is the major immunoreactive form of BNP in patients with heart failure. Clin. Chem..

[102] Semenov A.G., Postnikov A.B., Tamm N.N., Seferian K.R., Karpova N.S., Bloshchitsyna M.N., Koshkina E.V., Krasnoselsky M.I., Serebryanaya D.V., Katrukha A.G. (2009). Processing of pro-brain natriuretic peptide is suppressed by O-glycosylation in the region close to the cleavage site. Clin. Chem..

[103] Leduc R. (1993). Characterization of a secreted form of human furin endoprotease. Biochem. Biophys. Res. Commun..

[104] Dong N., Chen S., Yang J., He L., Liu P., Zheng D., Li L., Zhou Y., Ruan C., Plow E. (2010). Plasma soluble corin in patients with heart failure. Circ. Heart Fail..

[105] Fu S., Ping P., Zhu Q., Ye P., Luo L. (2018). Brain natriuretic peptide and its biochemical, analytical, and clinical issues in heart failure: A narrative review. Front. Phys..

[106] Dos Santos L., Salles T.A., Arruda-Junior D.F., Campos L.C., Pereira A.C., Barreto A.L., Antonio E.L., Mansur A.J., Tucci P.J., Krieger J.E. (2013). Circulating dipeptidyl peptidase IV activity correlates with cardiac dysfunction in human and experimental heart failure. Circ. Heart Fail..

[107] Gomez N., Touihri K., Matheeussen V., Mendes Da Costa A., Mahmoudabady M., Mathieu M., Baerts L., Peace A., Lybaert P., Scharpé S. (2012). Dipeptidyl peptidase IV inhibition improves cardiorenal function in overpacing-induced heart failure. Eur. J. Heart Fail..

[108] Talha S., Charloux A., Piquard F., Geny B. (2017). Brain natriuretic peptide and right heart dysfunction after heart transplantation. Clin. Transplant..

[109] Van der Meer P., Gaggin H.K., Dec G.W. (2019). ACC/AHA Versus ESC Guidelines on Heart Failure: JACC Guideline Comparison. J. Am. Coll. Cardiol..

[110] Wang T., Liu J., McDonald C., Lupino K., Zhai X., Wilkins B.J., Hakonarson H., Pei L. (2017). GDF15 is a heart-derived hormone that regulates body growth. EMBO Mol. Med..

[111] Dalos D., Spinka G., Schneider M., Wernly B., Paar V., Hoppe U., Litschauer B., Strametz-Juranek J., Sponder M. (2019). New cardiovascular biomarkers in ischemic heart disease-GDF-15, a probable predictor for ejection fraction. J. Clin. Med..

[112] Moghtadaei M., Polina I., Rose R.A. (2016). Electrophysiological effects of natriuretic peptides in the heart are mediated by multiple receptor subtypes. Prog. Biophys. Mol. Biol..

[113] Kim G.E., Kass D.A. (2017). Cardiac phosphodiesterases and their modulation for treating heart disease. Handb. Exp. Pharmacol..

[114] Cruickshank J.M. (1993). Phosphodiesterase III inhibitors: Long-term risks and short-term benefits. Cardiovasc. Drugs Ther..

[115] Muller B., Lugnier C., Stoclet J.-C. (1990). Involvement of rolipram-sensitive cyclic AMP phosphodiesterase in the regulation of cardiac contraction. J. Cardiovasc. Pharmacol..

[116] Abi-Gerges A., Richter W., Lefebvre F., Mateo P., Varin A., Heymes C., Samuel J.L., Lugnier C., Conti M., Fischmeister R. (2009). Decreased expression and activity of cAMP phosphodiesterases in cardiac hypertrophy and its impact on beta-adrenergic cAMP signals. Circ. Res..

[117] Mokni W., Keravis T., Etienne-Selloum N., Walter A., Kane M.O., Schini-Kerth V.B., Lugnier C. (2010). Concerted regulation of cGMP and cAMP phosphodiesterases in early cardiac hypertrophy induced by angiotensin II. PLoS ONE.

[118] Molina C.E. (2012). Cyclic Adenosine monophosphate phosphodiesterase type 4 protects against atrial arrhythmias. J. Am. Coll. Cardiol..

[119] Nagendran J., Archer S.L., Soliman D., Gurtu V., Moudgil R., Haromy A., St Aubin C., Webster L., Rebeyka I.M., Ross D.B. (2007). Phosphodiesterase type 5 is highly expressed in the hypertrophied human right ventricle, and acute inhibition of phosphodiesterase type 5 improves contractility. Circulation.

[120] Yanaka N., Kurosawa Y., Minami K., Kawai E., Omori K. (2003). cGMP-phosphodiesterase activity is up-regulated in response to pressure overload of rat ventricles. Biosci. Biochem..

[121] Mehel H., Emons J., Vettel C., Wittköpper K., Seppelt D., Dewenter M., Lutz S., Sossalla S., Maier L.S., Lechêne P. (2013). Phosphodiesterase-2 is up-regulated in human failing hearts and blunts β-adrenergic responses in cardiomyocytes. J. Am. Coll. Cardiol..

[122] Zoccarato A., Surdo N.C., Aronsen J.M., Fields L.A., Mancuso L., Dodoni G., Stangherlin A., Livie C., Jiang H., Sin Y.Y. (2015). Cardiac hypertrophy is inhibited by a local pool of cAMP regulated by phosphodiesterase 2. Circ. Res..

[123] Wagner M., Mehel H., Fischmeister R., El-Armouche A. (2016). Phosphodiesterase 2: Anti-adrenergic friend or hypertrophic foe in heart disease?. Naunyn Schmiedebergs Arch. Pharmacol..

[124] Kim D., Rybalkin S.D., Pi X., Wang Y., Zhang C., Munzel T., Beavo J.A., Berk B.C., Yan C. (2001). Upregulation of phosphodiesterase 1A1 expression is associated with the development of nitrate tolerance. Circulation.

[125] Rybalkin S.D., Rybalkina I., Beavo J.A., Bornfeldt K.E. (2002). Cyclic nucleotide phosphodiesterase 1C promotes human arterial smooth muscle cell proliferation. Circ. Res..

[126] Miller C.L., Oikawa M., Cai Y., Wojtovich A.P., Nagel D.J., Xu X., Xu H., Florio V., Rybalkin S.D., Beavo J.A. (2009). Role of Ca2+/calmodulin-stimulated cyclic nucleotide phosphodiesterase 1 in mediating cardiomyocyte hypertrophy. Circ. Res..

